# A qualitative study exploring the experiences of individuals living with stroke and their caregivers with community-based poststroke services: A critical need for action

**DOI:** 10.1371/journal.pone.0275673

**Published:** 2022-10-10

**Authors:** Hardeep Singh, Tram Nguyen, Shoshana Hahn-Goldberg, Samantha Lewis-Fung, Suzanne Smith-Bayley, Michelle L. A. Nelson

**Affiliations:** 1 Department of Occupational Science & Occupational Therapy, Temerty Faculty of Medicine, University of Toronto, Toronto, Ontario, Canada; 2 KITE Toronto Rehabilitation Institute-University Health Network, Toronto, Ontario, Canada; 3 Rehabilitation Sciences Institute, Temerty Faculty of Medicine, University of Toronto, Toronto, Ontario, Canada; 4 March of Dimes Canada, Toronto, Ontario, Canada; 5 Institute of Health Policy, Management and Evaluation, Dalla Lana School of Public Health, University of Toronto, Toronto, Ontario, Canada; 6 OpenLab, University Health Network, Toronto, Ontario, Canada; 7 Leslie Dan Faculty of Pharmacy, University of Toronto, Toronto, Ontario, Canada; 8 Bridgepoint Collaboratory for Research & Innovation, Sinai Health System, Toronto, Ontario, Canada; Universita degli Studi della Campania Luigi Vanvitelli, ITALY

## Abstract

**Background:**

Unmet poststroke service needs are common among people living in the community. Community-Based Stroke Services (CBSS) have the potential to address these unmet needs, yet there are no comprehensive guidelines to inform the design of CBSS, and they remain an understudied aspect of stroke care. This study aimed to describe the perceived barriers to accessing community-based stroke services, benefits from these programs and opportunities to address unmet needs.

**Methods:**

This was a qualitative descriptive study with interviews and focus groups conducted with people living with stroke and caregivers. Data were transcribed and analyzed thematically.

**Results:**

Eighty-five individuals with stroke and caregivers participated. Four key overarching themes were identified: facilitators and barriers to accessing and participating in community-based stroke services; components of helpful and unhelpful stroke services; perceived benefits of community-based stroke services; and opportunities to address unmet stroke service needs.

**Interpretations:**

The findings resonate with and extend prior literature, suggesting a critical need for personalized and tailored stroke services to address persistent unmet needs. We call on relevant stakeholders, such as policymakers, providers, and researchers, to move these insights into action through comprehensive guidelines, practice standards and interventions to personalize and tailor CBSS.

## Introduction

Stroke is a common cause of disability in Canada, with a growing incidence [1]. A stroke can cause long-term functional and psychosocial impacts, making the resumption of community living a challenge [2–4]. A 2011 American study found that up to 59% of people had unmet long-term clinical and social needs after stroke (e.g. a lack of information regarding stroke or loss of work activities) [5]. Similarly, an Australian study reported that two years after stroke, up to 84% of people reported unmet needs related to stroke information, physical and mental health, and return to work [6]. The prevalence of unmet needs is highest between six months to two years poststroke, but people can have long-term unmet needs 15 years after their stroke [5, 7]. Yet, in Canada, the median length of inpatient rehabilitation stay is only 35 days [8], and about 35% of people with stroke are discharged home from inpatient rehabilitation without further rehabilitation services [9]. Thus, community-based stroke services (CBSS) can be the only service option available for many individuals, particularly in the chronic stage, which extends multiple years poststroke.

CBSS can be loosely defined as any intervention, program, or service that assists people impacted by stroke with community reintegration. CBSS aim to maximize individuals’ potential to live with any lasting physical, cognitive, or mental health impacts, may help address unmet needs [10–14]. While CBSS in Canada have evolved considerably since the 1990s [15, 16], there remain knowledge gaps and unmet stroke service needs, particularly within CBSS [5–7]. Canadian Stroke Best Practices recommend that individuals with stroke and caregivers should have access to CBSS [17]. However, there are no standards to guide CBSS, which contributes to their breadth and variability [7]. For example, CBSS can include interventions to reduce social isolation, support psychosocial health and wellbeing and improve physical health (e.g., peer or caregiver support and recreation programs) and may or may not be delivered by a healthcare professional [7, 13]. Although many CBSS pivoted to virtual modes of delivery during the COVID-19 pandemic, demonstrating the potential to support the digitalization of stroke services [18, 19], there is a lack of guidance on virtual delivery of CBSS [20–23]. Given Canada’s aging population [24] and increasing stroke prevalence [1], increased evidence and access to effective CBSS is urgently needed [15, 25].

Virtual delivery of stroke health interventions has become increasingly popular [26, 27]. Although many CBSS pivoted to virtual modes of delivery during the COVID-19 pandemic, demonstrating potential to support the digitalization of stroke services [18, 19], there is a lack of guidance on the virtual delivery of CBSS [20–23].

CBSS may also benefit the health system by augmenting hospital-based stroke services [13, 14, 28, 29]. However, healthcare providers often overlook CBSS, such as stroke recovery groups, while individuals impacted by stroke perceive these as valuable stroke recovery services [30]. More research is needed into this underserved aspect of stroke care, considering the potential value of CBSS and the persisting variability and gaps in the provision of community-based services [5, 7, 31].

Insights from participants’ perspectives and needs are essential to informing the development of relevant and client-centred stroke services [5, 6, 32, 33]. Qualitative research captures valuable insights from individuals with lived experiences to inform improved access, outcomes and quality of stroke services [34]. Thus, this qualitative study aimed to gain insights into the perspectives of people living with stroke and caregivers on community poststroke services, as this is an under-explored aspect of stroke care [7]. Specifically, we aimed to describe facilitators and barriers to accessing CBSS, perceived benefits from these programs, and opportunities to address unmet stroke service needs.

## Methods

### Design

This study was part of a larger ‘*Community Stroke Perspectives*’ project. An exploratory, descriptive qualitative study design allowed us to meet study aims intending to generate insights from the experiences of individuals who have used these programs. In line with this approach, we strived to remain “close to the surface of the data and events” to generate descriptions that closely reflected participants’ perspectives [35, 36]. Ethics approval for this study was received from the research ethics board of Sinai Health System (MSH REB 21-0162-E). Verbal consent was obtained from participants at the time of data collection. The Standards for Reporting Qualitative Research checklist was followed to ensure comprehensive and transparent reporting [37].

### Participants and context

Individuals who were ≥18 years of age, had any type of stroke or were a caregiver or family member of someone with a stroke, and lived in the community were eligible to participate. We strived to recruit a diverse group of participants based on their location (rural/urban), stage of stroke recovery, sex, age and ethnicity. Participants were recruited using advertisements shared on social media and within stroke programs at March of Dimes Canada (a community-based organization). In addition, we asked participants to share the advertisements with others in their network who may be eligible.

### Data collection

Interviews and focus groups were conducted on Zoom or telephone by SLF (female Research Coordinator), SHG (female Scientist), and two other female Research Coordinators with qualitative research experience. The interviewers did not have any contact with participants prior to this study. A semi-structured interview guide (Table 1) facilitated the interview and focus group discussions. The guide was developed and pilot-tested with a group of people with lived stroke experience to ensure appropriateness and clarity of interview questions. Field notes were created by the interviewers during/after the interviews and focus groups on participants’ non-verbal communication (e.g. tone of voice, gestures) and interviews’ emerging insights [38]. Individuals with aphasia were included using aphasia-friendly communication techniques (e.g. PowerPoint slides of short questions, visual response options, including caregivers in the conversation). In addition, flexible asynchronous data collection (e.g. responding to interview questions via email or text) was an option for participants who could not attend the interview or focus group or had difficulties with verbal communication [39].

**Table 1:** Sample interview questions for interview guide.

Sample interview question	Examples of probes
Can you tell me a bit about the programs or services you have found the most helpful for you during your recovery journey (for caregivers—helpful for you during your loved one’s recovery journey)?	How about during your/their transition from the hospital or rehab to home? After your/their transition to home? During later stages of your/their recovery? While you/they were in the hospital?
	What services/services made this easier for you?
	Can you tell me a bit more about what services or services helped you achieve this?
	If another stroke survivor/caregiver was interested in this program/service, what would you tell them about it?
	Have any services or programs stood out in particular to you in terms of helpfulness? What made the service helpful?
	Can you tell me a bit about your greatest successes in the stroke recovery journey?
	Can you tell me a bit about any services or services that gave you encouragement throughout the journey? Can you tell me more about how they helped?
Can you tell me a bit about where you go for information on stroke, services, services and tools?	Are there specific people you connect with or places you go to look online or otherwise?
	How about during the transition to home? After the transition to home? In the hospital?
	What services/services have helped you to find information?
	Can you tell me a bit about what you think would make finding the information you need easier?
	Phone, group, internet, website, service, other?
Can you tell me about what programs or services were missing throughout the recovery journey? Things you wish you had access to but didn’t?	Can you tell me a bit about how you have addressed these challenges?
	What would have made facing these challenges easier for you?
	Can you tell me a bit about any other areas in your life where you need extra support?
Can you tell me a bit about how the services you received or programs you participated in suited your unique needs?	Appropriateness and tailoring to situation/culture/language/age?
	Did you find the activities and materials relevant to you?
	Was what you worked on useful for you?
	Was there educational information that was relevant to you?
	What was missing?
	What do you wish health care providers knew about your unique experience with stroke/being a loved one of a stroke survivor so that they could provide better services?

### Data analysis

Data were transcribed and then analyzed descriptively using a six phase inductive thematic analysis [40], which aligned with a qualitative descriptive approach [41]. In the first phase, HS (a female Assistant Professor and occupational therapist experienced in qualitative research) and TN (a female researcher experienced in qualitative research) familiarized themselves with the data by noting initial ideas while reading the transcripts and field notes. In the second phase, HS and TN independently created initial data-driven codes for small segments of data on Nvivo 12 (a qualitative data coding software). In the third phase, HS and TN independently identified themes within the data based on patterns noted within the initial codes. In the fourth phase, HS and TN compared their interpretation of the themes to ensure no relevant themes were overlooked and the themes aligned with the research aims [42]. In the fifth phase, the theme titles were redefined to ensure the essence of the content within the theme was captured and the themes were verified by SLF, SHG and MLAN (a female Scientist, Assistant Professor and Chief Knowledge Officer at March of Dimes Canada). In the final phase of the analysis, we reported the themes using data extracts to provide examples directly in the participants’ words.

## Results

A total of 85 participants (individuals with stroke and caregivers) participated in this study through individual interviews or focus groups between June and August 2021.

### Description of individual interview participants

Demographics of participants who completed individual interviews are presented in Table 2. Of the 30 individual interview participants, eight participants were caregivers, and 22 were individuals with stroke.

**Table 2:** Individual interviews.

Code	Sex	Age Range (years)	Person with stroke (PWS) or Caregiver	Years post stroke	Interview Format	Canadian Province	Urban/Rural
1	female	40–59	PWS	5–10	interview	Alberta	Urban
2	female	40–59	PWS	<10	interview	Ontario	Urban
3	female	40–59	PWS	5–10	interview	Alberta	Rural
4	male	60–80	PWS	5–10	interview	Nova Scotia	Urban
5	female	60–80	caregiver	5–10	interview	Nova Scotia	Urban
6	male	40–59	caregiver	3–5	asynchronous	Alberta	Urban
7	female	60–80	PWS	<10	asynchronous	Alberta and Ontario	Urban
8	female	40–59	PWS	5–10	interview	Ontario	Urban
9	female	18–39	PWS	>1	asynchronous	Alberta	Urban
10	female	18–39	PWS	3–5	interview	Saskatchewan	Urban
11	female	40–59	PWS	5–10	interview (caregiver present)	Ontario	Urban
12	female	18–39	PWS	>10	interview	Ontario	Urban
13	female	40–59	PWS	1–3	interview	British Columbia	Rural
14	female	60–80	PWS	3–5	interview	Ontario	Urban
15	male	60–80	PWS	3–5	interview	Ontario	Urban
16	female	40–59	PWS	5–10	interview	Ontario	Urban
17	male	60–80	PWS	5–10	asynchronous	Ontario	Urban
18	female	18–39	caregiver	1–3	interview	British Columbia	Urban
19	female	>80	PWS	3–5	interview	Nova Scotia	Rural
20	male	60–80	PWS	5–10	interview (caregiver present)	Ontario	Rural
21	female	60–80	caregiver	5–10	interview	Ontario	Rural
22	female	60–80	caregiver	>10	interview	Ontario	Urban
23	female	40–59	caregiver	1–3	interview	Ontario	Rural
24	male	60–80	PWS	>10	interview	Ontario	Urban
25	male	unknown	PWS	>10	asynchronous	Ontario	Rural
26	female	18–39	caregiver	3–5	asynchronous	Ontario	Urban
27	female	18–39	PWS	1–3	interview	Newfoundland and Labrador	Urban
28	female	>80	caregiver	1–3	interview	Ontario	Rural
29	female	60–80	PWS	>10	asynchronous	British Columbia	Urban
30	male	60–80	PWS	5–10	asynchronous	Ontario	Urban

#### Description of focus groups participants

The first focus group was conducted in English, with participants attending a peer-support group (n = 40 people with stroke; 50% male and 50% female); this focus group was split into three smaller breakout rooms, each with a different facilitator. The second focus group was conducted in English and Punjabi (via interpreter) with participants who attended a South Asian stroke program (n = 2 caregivers, 3 individuals with stroke; 4 females and 1 male). The last focus group was conducted in English, Mandarin and Cantonese (via interpreters) with participants who attended a Chinese language stroke program (n = 1 caregiver, 9 individuals living with stroke; 4 females and 6 males).

### Description of stroke services accessed

Participants described various CBSS they currently accessed or had accessed in the past, broadly categorized as psychosocial, functional or physical, and informational (Table 3). These services were delivered through various formats, including social media, physical booklets, webinars, or in-person (pre-COVID-19 pandemic).

**Table 3:** Examples of community-based stroke services accessed by participants.

*Types of community-based stroke services accessed*	*Examples of programs*
**Psychosocial**	Peer-support (including age-specific support groups), psychotherapy, recreational/leisure activities, culturally-tailored
**Functional or physical**	Exercise, aphasia supports, rehabilitation (e.g. occupational and physical therapy)
**Informational**	Stroke care navigation, understanding stroke, poststroke symptoms

Four themes were identified from the interviews and focus groups (see Table 4), described in detail below.

**Table 4:** Themes and subthemes.

Themes	Subthemes
Theme 1: Facilitators and barriers to accessing and participating in Community -Based Stroke Services (CBSS)	Subtheme 1a: Facilitators to accessing and participating in CBSS
	Subtheme 1b: Barriers to accessing and participating in CBSS
Theme 2: Components of helpful and unhelpful CBSS	Subtheme 2a: Components of helpful CBSS
	Subtheme 2b: Components of stroke services that are unhelpful
Theme 3: Perceived benefits CBSS	Subtheme 3a: Psychosocial benefits from participation in CBSS
	Subtheme 3b: Physical benefits from participation in CBSS
Theme 4: Opportunities to address unmet service needs	Subtheme 4a: Opportunities to enhance age-appropriate CBSS
	Subtheme 4b: Opportunities to enhance appropriateness of stroke informational services to their life and stroke recovery stage

### Theme 1: Facilitators and barriers to accessing and participating in CBSS

The first theme describes the facilitators and barriers to participants’ access and participation in CBSS.

#### Subtheme 1a: Facilitators to accessing and participating in CBSS

Participants discussed several factors that facilitated their access and participation in CBSS. For instance, a participant in the English focus group indicated that their registration in the community services was set up while they were still a hospital inpatient: “[staff member from the community] program came to see me in the hospital.” Others indicated that learning about available community services through reputable online sources (e.g. social media) facilitated their access to the services. Participants also mentioned a “snowball effect” wherein a new service user learns about other available stroke services through other members in the program as the new member “opens the first door and then meet others who open further doors.”

For individuals with sufficient digital literacy skills and access to a computer and the internet, the shift to virtual program delivery during the COVID-19 pandemic was described as a convenient and helpful way to access CBSS. In terms of factors that facilitated their participation in the program, one participant indicated that having services delivered in their primary language facilitated her participation: “at first [I] joined an English-speaking group and [my] English was not so well, so [I] didn’t understand some of the activities but now [I am] in a Chinese program.” Two participants indicated that offering the CBSS online facilitated their access to community support: “I’m grateful for virtual because the other option would be not connecting” (P3). Finally, participants indicated that a small, affordable fee or free membership facilitated their participation in CBSS.

#### Subtheme 1b: Barriers to accessing and participating in CBSS

Participants indicated no consistent or systematic ways they were referred to or accessed the CBSS; they were referred to these services through various sources, including friends and family, booklets, online self-referral, healthcare providers or another community program. Participants identified several barriers to accessing stroke services due to inconsistent referral mechanisms, including lack of awareness or delayed access to the programs. To mitigate this access barrier, P20 suggested that “the more services and support people are aware of, the better. These service organizations don’t seem to have a channel that tells people where to go.” Similarly, from a caregiver’s point of view, P26 shared her experience with a lack of awareness about available stroke services at a time they were necessary:

“It’s a very overwhelming process in the beginning and I wish there was greater emphasis on resources available much earlier on. I found that my mother was given *some* resources at a doctor’s appointment early on, but as a caregiver, I wish I had access to the same info she was receiving… If I had been made aware, I could have been a greater supporter of exploring this MUCH needed resource; there was definitely a lack of awareness.”

However, even when aware of the community services, not all participants reported being able to access them. Several participants explained that relying on others for transportation assistance was a significant access barrier, especially considering many people had their license suspended after a stroke. P14 explained that where they lived was another access barrier as community services were unavailable nearby:

“It’s pretty sad that there’s such a huge difference between the smaller cities and I have no idea why that is…I remember when I was researching, so many [services] popped up from out of town.”

A South Asian focus group participant who broke her hip at home twice and could not access CBSS explained that pre-existing or comorbid conditions were another barrier to accessing CBSS. Similarly, P3 alluded to accessibility barriers in the built environment of the community program restricted participation because “not everyone can walk.” However, while virtual programs were an effective solution to mobility and transportation barriers for some, they posed additional barriers for individuals who did not have the required resources and digital literacy skills (i.e. “not the most computer literate” P4). As P16 explained, “not everybody who has a stroke can connect virtually. I am so so with computers, but managed to feel my way through.” Additionally, P23, a caregiver, explained that the program’s schedule was an access barrier: “Would love to join caregiver groups, but all during the day and I have to work for the first time since he had his stroke so can’t do it now.” Finally, participants indicated that some community programs, particularly physical interventions (e.g. private physical therapy), were costly and financially inaccessible.

In sum, facilitators to access and participate in CBSS included increased awareness of and access to referral processes, programs available in their preferred language, offered online, and perhaps low or no cost. Barriers that reduced or prevented their access to the services included no consistent referral process to learn about these services, a lack of awareness, and logistical and technical barriers impacting access.

### Theme 2: Components of helpful and unhelpful CBSS

This theme captures participants’ perspectives on what components they considered were helpful and unhelpful within CBSS.

#### Subtheme 2a: Components of helpful CBSS

CBSS were considered helpful when they provided participants with relevant information. However, what participants considered relevant information differed based on their unique situations. For example, P24 explained that the content had to be geared toward each service user to be *relevant*: “everyone’s individualism has to be recognized. It’s difficult for some people to make that recognition and apply it for several survivors.”

Participants explained that the CBSS must create a compassionate space where they feel heard, motivated, and connected to others who share similar experiences and interests. For example, a participant in the South Asian group explained, “Since I started my program, I feel really supported and helped with the exercises we are doing and interacting with people in similar situations. I feel they are such a supportive program for me.” A similar sentiment was shared by a participant in the Chinese language group: “interacting with people in similar situations. I feel they are such a supportive program for me…[the program] helped [me] to have a more normal social life.” Similarly, younger individuals with stroke reported that being able to access services geared to the needs of younger adults (e.g., developing skills needed to return to work) and interacting with peers of a similar age was helpful.

#### Subtheme 2b: Components of stroke services that are unhelpful

Participants provided insights into what components of stroke services were unhelpful. Specifically, they explained that it was unhelpful when service providers overpromised the potential benefit of the program: “they promised you that you were going to be able to use your arm at the end of it” (P4). Participants also explained that poor communication and rapport with service providers negatively impacted their program use (e.g., if the service provider disagreed with them or their caregiver). In addition, they explained that while hospital-based stroke services were personalized and tailored to their needs, this was lacking in the community. P24 called these “cookie-cutter” programs (i.e. generic programs not tailored to participants’ needs): “The one thing that is probably the biggest no-no in any program or support is being a cookie-cutter program that should cover everyone…There are too many cookie cutters programs” (P24).

There were variable preferences regarding the delivery format of CBSS. While some participants preferred a group format within CBSS, others like P11 were uncomfortable with group classes: “I I like exercise [programs] but I don’t like being watched and stared at. There are a lot of people there but I don’t want to be judged.”

The timing of programs along their stroke recovery mattered to some participants as participants were at different stages of stroke recovery and had different needs for programmatic content. For instance, P16 explained that she was not ready to access stroke support in the early stages of her stroke:

“They gave me this book…I don’t want to read this. This doesn’t help me. I had my stroke eight days after giving birth to my daughter. I couldn’t feel anything. How can I do my exercises if I don’t feel?!”

In contrast, soon after the stroke, P24’s priority was to progress their physical recovery rather than receive social support:

“In this whole process, it’s always been *physical* recovery. It still is. I don’t spend any time on cognitive recovery…There are a number of programs related to after stroke. They just want to *share experiences*. They just want to be there, with other people.”

In sum, components considered helpful in CBSS included relevant content, creating a compassionate space, and connecting to others in similar situations. In contrast, unhelpful components of CBSS included overpromising the program’s benefits, poor delivery format (e.g. cookie-cutter approach), poor communication with the program staff, and the timing or goals of the program did not match their stage of stroke recovery.

### Theme 3: Perceived benefits CBSS

This theme describes participants’ perspectives on the psychosocial and physical benefits of participating in CBSS.

#### Subtheme 3a: Psychosocial benefits from participation in CBSS

Participants primarily discussed psychosocial benefits of CBSS. For example, one participant in the Chinese language group shared that the CBSS helped reduce depressive symptoms: “[I] used to have depression, but programs helped [me] to recover and have a normal social life.” Multiple participants cited enhanced socialization and reduced loneliness from CBSS. For instance, a participant in the first focus group explained, “[the stroke program] makes you feel like I’m not alone in this.” Additionally, it was “helpful to learn what other people are doing—compare, can I do that too? can I try?” Participants in the South Asian and Chinese language focus groups indicated that the social environment created by their peers and program staff within the CBSS helped them feel “really supported” and encouraged them to try new things: “At first, [I] couldn’t’ walk or move [my] fingers when [I] first joined the programs, but all the other participants encouraged [me] to try, so [I] didn’t give up. Now [I] can write words and walk.” Many participants indicated that the peer support offered within the CBSS helped them “sort out the good information from the bad.” In addition, one participant with stroke indicated that the community services helped with their hospital-to-home transition: “When I came home from the hospital, I felt so alone for weeks. I wasn’t connected to anyone, and it was very difficult to cope. This group helped me with ideas from sharing their experiences.”

#### Subtheme 3b: Physical benefits from participation in CBSS

Some participants described physical benefits from participating in CBSS, such as finding new ways to communicate and improving strength and fine motor skills. As P19 explained, “It’s really just exercise. It’s been really good. You can be lazy, so it’s good to have someone there and showing you.” P1 explained that a program tailored specifically to younger individuals with stroke was preferred as the activities were more stimulating: “The young stroke survivors were more my age, and they needed to get exercise, so that was more helpful and stimulating to me. We did things like bowling, yoga, assisted canoeing trip, and that was great” (P1).

In sum, participants described psychosocial benefits from participating in CBSS, such as encouragement, social support and motivation and reduced isolation and loneliness and physical benefits, such as enhancing communication skills and physical stimulation/exercise.

### Theme 4: Opportunities to address unmet service needs

This theme captures opportunities for personalization of CBSS, such as age and stage-appropriate resources.

#### Subtheme 4a: Opportunities to enhance age-appropriate CBSS

Participants explained that most services were tailored to older individuals with stroke, and there was a services gap for younger (<65 years of age) individuals with stroke who were in a different life stage. For example, P10 proposed the development of a support group for “working people who struggle with the same thing. I want peer support where I have same struggles…More resources for youth (under 50 years of age).” Another suggestion was to create employment opportunities or offer return-to-work training for younger adults with goals related to returning to work: “More employment opportunities. Training for something new since can’t do what he did before. [He] was a home contractor. Loved his job.” Lastly, participants stressed that stroke informational resources should be more appropriate for people of all ages: “Like a Stroke recovery handbook, for all ages. There is so much information on recovery for those over the age of 65, but not for young survivors. I wish it was easier for the Young” (P9).

#### Subtheme 4b: Opportunities to enhance appropriateness of stroke informational services to their life and stroke recovery stage

Considering the vast amount of information, participants desired the need for “reliable Canadian sources for information. Too much on Google and hard to sort the trash out” (P20 and P21). In particular, participants at the early stages post-stroke indicated feeling isolated and confused about the next steps after their hospital discharge due to a lack of navigation services.

“When I first had the stroke, there wasn’t anything really set up at all. Perhaps I got lost or feel through the cracks or something…Instead of the hospital sending me straight to inpatient rehab, for some reason that did not happen with me so they sent me home. So at that point I was in a wheelchair and unable to do much for myself or to do anything, so I was like what’s next, what’s going to happen? They gave me no information, no phone numbers to call” (P14).

Participants also indicated that it would be beneficial to have some “guidance” about what to expect after the acute phase of the stroke to improve their adjustment and community transition.

“The lack of information is the biggest one…my brain had to improve to some sort of level to get to that point. Perhaps two or 3 weeks after my stroke I could get to Toronto—that didn’t happen. It clicked eventually. The whole lack of research and information and resources. Someone should have been there. The support system should have been there for anyone who goes through this” (P1).

Informational needs depended on personal life circumstances (e.g. employment) and the stage of stroke recovery. Personalizing this information to the participant’s life circumstances and stage of recovery was recommended to enhance the appropriateness of CBSS. Depending on their stage of stroke recovery, participants had various informational needs, including how to deal with poststroke symptoms, such as fatigue, post-traumatic stress disorder and falls. Moreover, managing finances after a stroke were highly stressful, and financial guidance was noted as a current informational gap.

Peer support was described as an effective solution to increase access to information on stroke and what to expect. For instance, P19 explained,

“Dealing with insurance and CPP [Canadian Pension Plan]. It’s a very complex and legal regime of what insurance can and cannot demand of you. For people who have long term insurance, they’re being forced to do things by their insurance company when they can’t and they shouldn’t. You need someone who has experience in that area, not a lawyer but someone with lived experience. So I would say high-level peer support.”

Finally, family and caregiver services and education were highlighted as areas to further develop in CBSS:

“Education for the caregiver—how to deal with your loved one but also how to deal with your own fears. Have to take over “male” tasks. Maintenance on car. Everything done by my partner was my job to do for months and years…Where to find bank info, etc…anything that the partner was the one to do that all. What to expect? In some cases, the male partner has all of the financial information such as credit cards” (P20 and P21).

In sum, participants indicated the need for more personalization of information to their age and stage of recovery, and they made recommendations to enhance informational and navigation support provided by CBSS. Based on the study findings, we have generated practice recommendations for CBSS to address unmet stroke service needs (Table 5).

**Table 5:** Practice recommendations for community stroke services to enhance access and reach.

Service component	Practice recommendations for community-based stroke programs to enhance relevancy and impact
***Referrals***	• Create a more systematic referral to available stroke programs (e.g. during hospitalization) • Create multiple paths of referral to ensure users do not fall through the cracks
***Tailoring content***	• Balancing feasibility, resource constraints, and a wide variety of interests (e.g. group exercise, peer support, education, skills-based) while allowing for flexibility and tailoring to culture, language, stage of stroke recovery, and age
***Delivery format***	• Consider the location of programming (e.g. built-environment accessibility) • Explore the utility of virtual programs to remove barriers related to location, transportation, and health issues • Address access to resources (e.g. devices and wifi) and lack of digital literacy through digital literacy training and/or offering in-person options
***Language & communication***	• Offer programs in languages spoken by dominant minority language groups • Use lay language to address issues related to understanding of terminology • Program leaders should be sensitive to the differing opinions and needs of users • Set realistic expectations of the program’s benefits
***Appropriateness***	• Participants’ age, life stage, recovery stage, communication • Poststroke outcomes • Participants’ goals and interests • Culture/language

## Discussion

This qualitative study provides valuable insights into the experiences of 85 individuals with stroke and caregivers participating in CBSS in Canada. While the findings from this review highlight the valuable role of CBSS, they reveal a critical need to address the unmet service needs of individuals impacted by stroke through personalized and tailored CBSS.

Our study supports this conversation by describing insights from a large sample of participants regarding the value and benefits of community services in addressing their needs related to psychosocial wellbeing and information [14, 30]. Psychosocial needs such as depression, anxiety, and social isolation are concerns after stroke [43–45], and CBSS that offer peer support may support the psychosocial stroke recovery process by reducing isolation, creating community, and empowering people [46].

Information about stroke can improve patient and family’s stroke knowledge and decrease depression symptoms [47], and although considerable information about stroke exists, individuals with stroke and caregivers have reported a low understanding of stroke [47–49]. Our findings suggest that CBSS have the potential to address participants’ unmet informational needs. However, as previously mentioned, tailored information in multiple formats is needed to comprehensively address the diverse needs of individuals with stroke, including their stroke-specific impairments and individual circumstances [47]. In addition, our findings support the need to improve the referral process to CBSS [48] and the potential of virtual modes of delivery to meet the ongoing service needs of people who experience accessibility, travel or other access barriers [20, 50]. We also noted that increased access to CBSS through virtual delivery was reported not only by individuals residing in rural locations, but also those in urban areas, particularly by those with mobility restrictions and transportation barriers (e.g. individuals who had their license suspended after a stroke). However, future research may be needed to identify the effectiveness of virtual CBSS compared to in-person programs and optimal delivery mechanisms.

Overall, it is surprising that a number of the stroke service needs identified from our large sample size resonate with prior stroke literature, suggesting that they persist and remain unaddressed [4, 49, 51, 52]. Specifically, there is mounting evidence for *personalized* and *tailored* services (e.g. tailored to age, time since stroke, communication, culture) across the stroke care continuum. Despite a strong knowledge base of the benefits of personalized services, our study revealed that these needs persist due to a lack of personalized/individual stroke services available in the community [4, 49, 51, 53, 54]. Future research should investigate organizational considerations for program design to support personalized and tailored services. This problem may also reflect a more extensive implementation issue in integrating research evidence into practice [55, 56]. “Relevant stakeholders involvement” (e.g. end-users, policymakers, researchers and clinicians) is considered *essential* to integrating research evidence to practice, as they must collaboratively develop and implement solutions that align with the health system constraints to effectively address these persistent stroke service gaps and unmet needs [55–57]. Collaborative research methods, including community-based participatory research and integrated knowledge translation, which promote knowledge sharing between researchers and knowledge users, could be a starting point to bringing knowledge to action [57]; however, implementation efforts on a larger scale are required to impact stroke service delivery [55, 56].

### Limitations and strengths

The first limitation is that we recruited most participants through the network of a single organization. Second, we did not comprehensively capture participant characteristics (e.g. focus group participants’ age, time since stroke) or other identity factors shaping poststroke needs (e.g. socio-economic variables). Third, there was a limited representation of individuals with severe aphasia or cognitive poststroke challenges; their unique perspectives warrant further exploration. Fourth, we had limited representation from caregivers, which prevented a subgroup analysis. Finally, participants were primarily White and English-speaking; non-English speakers and ethnic minorities may have different experiences with community-based stroke programs and unmet stroke service needs that warrant further investigation [58].

Despite these limitations, these findings have the potential to inform future research and practice directions. This study was co-designed with a working group with lived stroke experience to capture meaningful insights. In addition, participants’ perspectives from across Canada were represented, including those residing in rural and urban locations. The study sample was large and included individuals at different stages poststroke, allowing us to identify differences in service needs based on stages of stroke recovery.

## Conclusions

In conclusion, participants described the benefits of participating in CBSS but also indicated unmet poststroke service needs related to a lack of personalization and tailoring of services. As these study findings resonate with prior literature from across the stroke service continuum, we contend that there is a critical need for stakeholders to develop and implement clear, pragmatic stroke service solutions collaboratively, such as comprehensive guidelines, practice standards and interventions for personalized and tailored CBSS.

## Supporting information

S1 File (DOCX)
